# Risk of Confusion: Detection of a Circular Thickness of the Wall in the Lower Part of the Esophagus

**DOI:** 10.4021/gr313w

**Published:** 2011-07-20

**Authors:** Achim Hochlehnert, Sylvie Lorenzen, Peter Hallscheidt, Jens Encke, Robert Ehehalt

**Affiliations:** a Department of General Internal and Psychosomatic Medicine, University of Heidelberg, Medical University Hospital, Heidelberg, Germany; b National Center for Tumor diseases (NCT), University of Heidelberg, Germany; c Department of Radiology, University of Heidelberg, Medical University Hospital, Heidelberg, Germany; d Johanna Etienne Klinik, Neuss, Germany; e Department of Gastroenterology, University of Heidelberg, Medical University Hospital, Heidelberg, Germany

**Keywords:** Carcinoma of the esophagus, CT scan, Endoscopy

## Abstract

We report a case of a woman with a gastrointestinal bleeding. An esophageal ulcer was detected in the endoscopy, however a histological malignancy could not be found. A computer tomography (CT) scan showed a thickness in the distal esophagus and enlarged lymph nodes, so a malignancy was highly suspected. However, the patient refused to follow the recommended clinical procedure of a surgical intervention. Four years later a carcinoma could be ruled out because of follow-up examinations.

## Case Report

A 60 year old lady with a long-time history of alcohol abuse was admitted to the hospital in December 2005. She had complained about increasing weakness for two weeks. Because of increasing dizziness she turned to her general practitioner who found a slight anemia due to iron deficiency and prescripted iron orally. After a collapse at home with a short loss of consciousness she was transferred to the neurological emergency room. After a short while she felt better and discharged herself on her own responsibility but immediately collapsed again in front of the door of the neurological department. She was then transferred to the emergency room of the Internal Hospital, where the laboratory results showed remarkably reduced hemoglobin (2.8 g/dL). Pathological laboratory findings included reduced spontaneous thromboplastin time (61%, INR 1.3) and an elevated tumor marker (CEA 3.7 µg/L, normal range: < 2.5 µg/L) (Table 1).

**Table 1:** Laboratory Findings on Admission

Parameter	Value	Norm
CEA	**3.7**	< 2.5 µg/L
Thromboplastin time	**61**	> 100%
PTT	21.3	ca. 28-48 sec.
Sodium	142	135-145 mmol/L
Potassium	4.12	3.5-5.0 mmol/L
Creatinine	0.75	-1.30 mg/dL
BUN	62	-45 mg/dL
Glucose	**130**	65-110 mg/dL
CK	27	-80 U/L
LDH	146	120-240 U/L
AST	10	-18 U/L
ALT	8	-24 U/L
ALP	30	40-170 U/L
γ - GT	30	3-28 U/L
CHE	**3.32**	3-9.3 KU/L
CRP	< 2.0	-5 mg/L
Amylase	23	- 110 U/L
Lipase	27	-190 U/L
Total Bilirubine	0.2	-1.0 mg/dL
Albumin	**27.6**	30-50 g/L
Haemoglobin	**2.8**	12-15 g/dL
Red cell count	**1.0**	4.0-5.2/pL
Hematocrit	**0.10**	36-47%
Platelets	296	150-440/nL
WBC	**12.37**	4.0-10.0/nL

pathological findings are formatted in bold.

**Figure 1:**
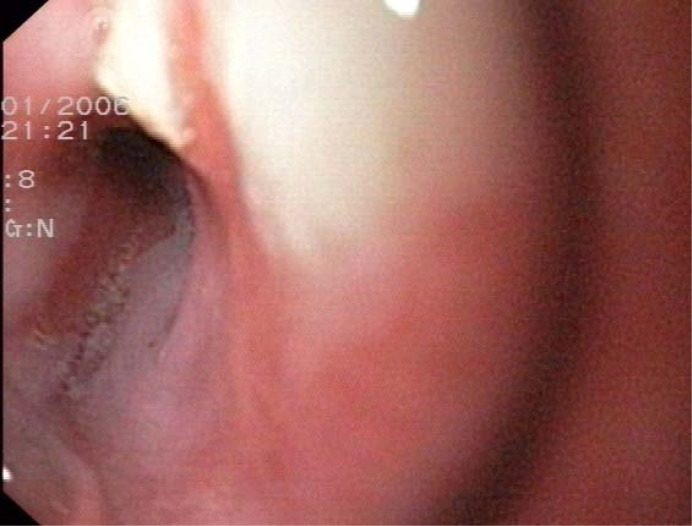
Esophageal ulcer.

She was directly admitted to the gastroenterological intermediate care unit (IMC) and was substituted with red blood packed cells. Although there were no clinical signs of GI- bleeding (melena or hematemesis) a gastroscopy was performed which detected an esophageal ulcer (Fig 1).

**Figure 2:**
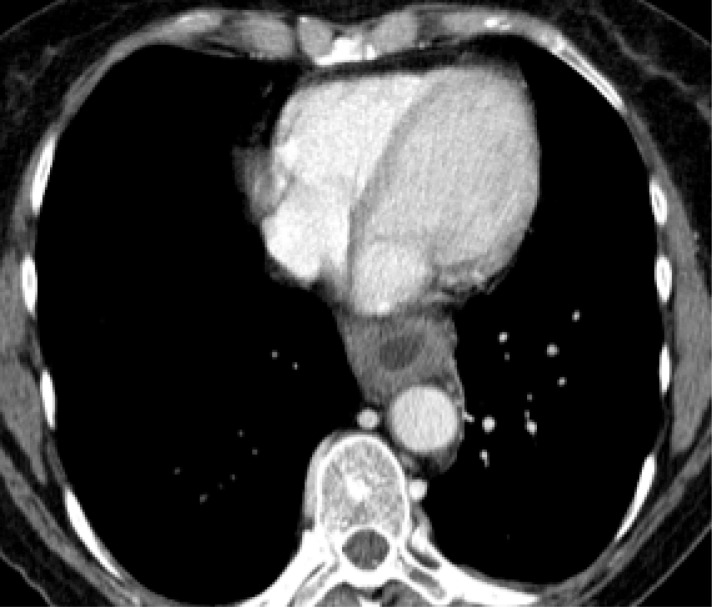
Circular thickness of the wall in the lower part of the esophagus and several augmented lymph nodes in the mediastinum and retroperitoneal.

Computed tomography (CT) scan of the thorax performed on the next day with a special esophageal protocol (water filling) confirmed the finding and showed a circular thickness of the wall in the lower part of the esophagus as well as several augmented lymph nodes in the mediastinum and retroperitoneal. Esophageal perforation or a pneumothorax could not be detected (Fig 2).

The patient went on high dose PPI treatment and was stable during 3 days of monitoring in the intermediate care unit. Consecutively, she was transferred to the regular ward. No further event of bleeding was detected for the duration of stay in hospital.

**Figure 3:**
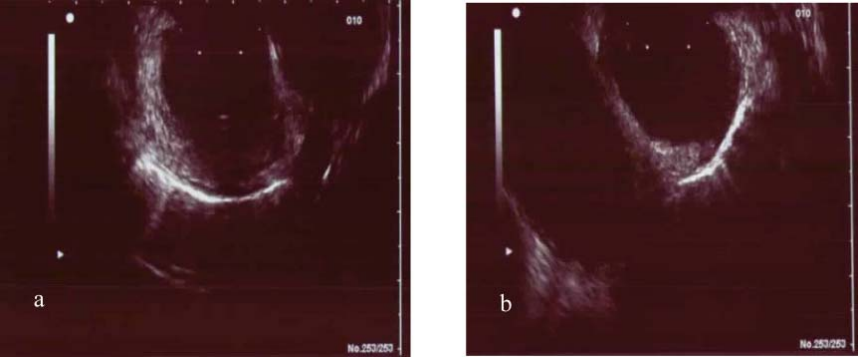
(a, b) Subtotal stenosis of the distal esophagus with exhaustion of the muscularis and several para-aortal lymph nodes.

Endoscopic ultrasound (EUS) examination described a subtotal stenosis of the distal esophagus with exhaustion of the muscularis and several para-aortal lymphnodes, which was suspicious for a carcinoma (Fig 3a, b).

To rule out an esophageal carcinoma several biopsies in the area of the suspected carcinoma were performed, showing no malignancy. The taking of the biopsies turned out to be difficult because the area of the suspected carcinoma was remarkably callous so the caliper glided off easily. All following histological examinations showed no malignancy, but a carcinoma of the esophagus was profoundly assumed and a surgical resection was recommended based on the control CT scan 8 weeks later, which showed an unchanged result. However, the patient refused to underlay this procedure. After discharge from the medical hospital the patient was readmitted three times because of recurrent upper gastrointestinal bleeding and reexamined in the further course: The follow up CT scans in September 2006, September 2007 and December 2009 showed unaltered findings. Consequently, the initial diagnose of an esophageal malignancy turned out to be wrong. The surgical resection would have included a complicated procedure with a high morbidity and mortality [1, 2].

## Discussion

The present case report focuses on the discrepancy between a highly suspected carcinoma by radiological and endoscopic findings on the one and no histopathological proof of malignancy on the other side.

Our case shows as well that, if chronically, an upper GI-bleedings may sustain undetected for a long time. In this case a collapse was the first symptom to be admitted to a hospital which is per se not so much suggestive. In general, dysphagia, pain and weight loss are more common reasons for patients to turn to a medical department for this diagnosis [3]. Although no clinical signs of active bleeding (melena, hematemesis) were present, an upper GI endoscopy was perfomed that showed an esophagus ulcer.

Since esophageal cancer is a common cause of esophageal ulcer the lesion was biopsied several times to enforce a histopathologic diagnosis of cancer. However, repeated endoscopic examinations did not lead to a final diagnosis of cancer. Literature shows a wider range of sensitivity (69% versus 100%) mainly depending on the experience of the endoscopists [4]. Therefore a CT of the chest with esophageal protocol was performed (We recommend a special protocol for the esophagus with double contrast. This protocol has been developed in the Radiological Department at the University of Heidelberg and is often performed with a precise benefit of information compared to a normal CT scan of the thorax. It is characterized by a distension of the wall of the esophagus due to the filling of the esophagus with water, which allows a better appraisal. Concurrently, the treatment with butylscopolamine is performed to eliminate the peristaltic, so with a modern spiral CT the appraisal of the whole esophagus can be enabled within only a few seconds. A reconstructed slice thickness of 4 mm is performed in a venous phase with a delay of 80 seconds.). This allows panoramic exploration, virtual endoluminal visualization, accurate longitudinal and axial evaluations and simultaneous evaluation of T and N parameters [5-8]. CT also provides a better presentation of the mucosa, and an appraisal of the surrounding tissue of the lumen beside the oesophagus. A study with multidetector CT showed that 2 of 16 esophageal cancers remained undetected, so sensitivity could be specified as 87.5% [9].

In our patient, the CT scan was suspicious of an invasive tumor. In addition CEA was slightly elevated, and an endosonografic examination confirmed this finding.

Adenocarcinomas are the most dominant subtype of proven malignancies in the distal esophagus [3]. Rarely gastrointestinal stromal tumours (GIST) occur in the esophagus, especially in these cases CT can help identify the tumour and assess for local spread or distant metastasis [10]. In our case enforced histological examinations showed no malignant result, however, based on the CT scan a carcinoma of the esophagus was profoundly assumed. Due to the distension of the lesion, a surgical approach was recommended after an interdisciplinary discussion. Radiotherapy was not favoured because of the augmented lymph nodes. A neoadjuvant therapy which could have been an alternative because of the suspected lymph nodes was not taken into closer consideration.

Esophageal and gastric cancers are distinct carcinomas of the upper gastrointestinal tract, while the distinction between them was less clear at the gastroesophageal junction at time of first presentation 2005 [11]. Meanwhile the present classification provides a distinct allocation [12].

Although the possible existence of micrometastasis in superficial esophageal cancer is the most important factor in deciding the therapeutic strategy, it is difficult and molecular biomarkers are not a valuable selection at this stage [13]. The most complete preoperative staging for patients with esophago-gastric cancer is a complementary manner of CT, endoscopic ultrasound (EUS) and probably laparoscopic ultrasound [14], though these investigations precluded resection in about one-third of patients with adenocarcinoma of the gastro-esophageal junction [15].

Further radiological imaging (MRI, PET) was not performed in our case, because the diagnostic value was contradictory discussed at time of first presentation. However, high-resolution MRI has a high diagnostic accuracy for evaluating mural invasion [16] and esophageal adenocarcinoma could be clearly distinguished with magnetic resonance spectroscopy even in histologically indistinguishable tissue [17]. Beside an increased choline-to-creatine ratio a relative decrease in the carbohydrate region has been reported [18]. PET scan might be an alternative, because it permits a better detection of distant metastasis although the sensitivity of PET for loco regional lymph nodes is low [19]. In comparison to CT and EUS the diagnostic value of PET in the staging of adenocarcinoma of the esophagus and the esophago-gastric junction is limited because of low accuracy in staging of paratumoral and distant lymph nodes [20].

### Conclusion

Non neoplastic esophageal ulcers are a rare cause of recurrent GI-bleedings. CT scan with a special esophageal protocol is an important tool for differential diagnostic considerations. However, its sensitivity is not 100% so it does not reveal all patients with esophageal cancer successfully. Therefore the histopathological proof of neoplasma seems essential because of the high perioperative morbidity and mortality. Our case shows that there might be a risk of confusion because a circular thickness of the wall could be misinterpreted as a malignant lesion. If newer diagnostic tools like PET scan will help to better differentiate between malignant and non malignant lesions has to be shown.
